# Significant Decrease in Annual Cancer Diagnoses in Spain during the COVID-19 Pandemic: A Real-Data Study

**DOI:** 10.3390/cancers13133215

**Published:** 2021-06-28

**Authors:** Sofía Ruiz-Medina, Silvia Gil, Begoña Jimenez, Pablo Rodriguez-Brazzarola, Tamara Diaz-Redondo, Mireya Cazorla, Marta Muñoz-Ayllon, Inmaculada Ramos, Carmen Reyna, María José Bermejo, Ana Godoy, Esperanza Torres, Manuel Cobo, Laura Galvez, Antonio Rueda, Emilio Alba, Nuria Ribelles

**Affiliations:** 1 Medical Oncology Intercenter Unit, Regional and Virgen de la Victoria University Hospitals, IBIMA, 29010 Málaga, Spain; sofiaruizmedina@gmail.com (S.R.-M.); silviagilcalle@yahoo.es (S.G.); begojrodriguez@gmail.com (B.J.); tdredondo@gmail.com (T.D.-R.); mirecalo@hotmail.com (M.C.); ayllon.m@gmail.com (M.M.-A.); ramosgarcia22@gmail.com (I.R.); c.reyna.fortes@gmail.com (C.R.); cheberpe@gmail.com (M.J.B.); anagodort@gmail.com (A.G.); esp_torres2001@yahoo.es (E.T.); manuelcobodols@yahoo.es (M.C.); lauragalvezcarvajal@hotmail.com (L.G.); antonio.rueda.dominguez@juntadeandalucia.es (A.R.); ealbac@uma.es (E.A.); 2 Grupo de Inteligencia Computacional en Biomedicina, ETSI Ingeniería Informática, Universidad de Málaga, 29071 Málaga, Spain; pabrod@uma.es

**Keywords:** cancer diagnoses, COVID-19 pandemic, real data

## Abstract

**Simple Summary:**

There is no doubt about the transformation that health systems have undergone in response to the COVID-19 pandemic, but this may have had a negative impact on other diseases such as cancer. We have analyzed all the cancer patients diagnosed in the first post-pandemic year in two university hospitals that attend 785,000 people and compared them with patients diagnosed in 2019. Our results clearly show that during the first post-pandemic year there has been a significant decrease of 17.2% in the number of patients diagnosed with cancer. However, there were no differences in the number of patients diagnosed between 2018 and 2019 or between 2017 and 2019, so that loss of cancer patients could be attributed to changes made in health systems due to the pandemic. The delay in the diagnosis may have important consequences in the prognosis of cancer patients, so health systems should carry out an effort similar to that made with the pandemic to recover all lost patients who have remained undiagnosed.

**Abstract:**

The COVID-19 pandemic has caused a profound change in health organizations at both the primary and hospital care levels. This cross-sectional study aims to investigate the impact of the COVID-19 pandemic in the annual rate of new cancer diagnosis in two university-affiliated hospitals. This study includes all the patients with a pathological diagnosis of cancer attended in two hospitals in Málaga (Spain) during the first year of pandemic. This study population was compared with the patients diagnosed during the previous year 2019. To analyze whether the possible differences in the annual rate of diagnoses were due to the pandemic or to other causes, the patients diagnosed during 2018 and 2017 were also compared. There were 2340 new cancer diagnosis compared to 2825 patients in 2019 which represented a decrease of −17.2% (*p* = 0.0001). Differences in the number of cancer patients diagnosed between 2018 and 2019 (2840 new cases; 0.5% increase) or 2017 and 2019 (2909 new cases; 3% increase) were not statistically significant. The highest number of patients lost from diagnosis in 2020 was in breast cancer (−26.1%), colorectal neoplasms (−16.9%), and head and neck tumors (−19.8%). The study of incidence rates throughout the first year of the COVID-19 pandemic shows that the diagnosis of new cancer patients has been significantly impaired. Health systems must take the necessary measures to restore pre-pandemic diagnostic procedures and to recover lost patients who have not been diagnosed.

## 1. Introduction

There is no doubt that the COVID-19 pandemic has transformed healthcare like no other emergency has done before. In a short time, it has been necessary to allocate the use of a large part of the health services resources to the care of COVID-19 patients, which has had a notorious impact on the management of other diseases. 

Changes have occurred at all levels of care, with data indicating a 30% decrease in primary care consultations, of which more than half have been carried out in a remote mode [1], and the temporary closure of >60% of hospital outpatient radiology facilities [2]. In addition, another factor to consider in the delay of the screening, diagnosis, or treatment of non-COVID-19 diseases has been the patients themselves, because they have deferred or suspended visits to health facilities for fear of contracting the coronavirus [3,4,5].

Cancer patients have also been affected by the decrease in health resources designated to their management. The authorities of several countries gave instructions to temporarily suspend breast, colorectal, and cervical cancer screening programs [6,7]. Medicare data show that screening procedures for breast, colon, prostate, and lung cancer were lower in March–July 2020 compared to March–July 2019, with a maximum decrease in April of 85%, 75%, 74% and 56%, respectively [8]. Breast and colorectal screening encounters declined by 89.2% and 84.5%, respectively, between January and April 2020 compared to the same period in 2019, according to data from the TriNetX platform [9]. 

A decrease in the number of new diagnosed cancer cases has been reported by authors from several countries. Data from US patients showed that during March-April 2020, the weekly number of newly identified patients with breast, colorectal, lung, pancreatic, gastric, or esophageal cancer decreased by 46.4% compared to January 2019-February 2020 [10]. In the Netherlands, the number of new weekly cases dropped by 26% in April compared to January 2020 [6]. Also, a decline of 44.9% in weeks 11 to 20 of 2020 compared to 2018 to 2019 was found in Italy [11] and the number of new cancer diagnoses fell by 30% in April 2020 compared to April 2019 in Germany [12]. Furthermore, a significant increase in the incidence of more advanced stages of melanoma [13], breast [14,15] and lung cancer cases [16] at diagnosis has been observed.

Likewise, cancer treatments have been affected by the pandemic. According to data from the National Health Service (NHS), in England, there was a relative reduction of 22% in the number of diagnosed cases of colorectal cancer that were included in the 31-day-to treatment pathway, and of 31% in the number of colorectal cancer operations in April 2020 compared with 2019 [17]. In the same period, the number of radiotherapy courses delivered across the English NHS fell by 20% [18]. Between March to July 2020, a decrease in the billing frequency for chemotherapy administration was observed in comparison to 2019, with a maximum of 32% in July, according to Medicare data [8]. Data from a multicenter nationwide study from Spain that collected information from departments treating oncology patients through an electronic questionnaire, reported a decrease of 20.3% in the number of new patients’ referrals and of 14.3% in the mean number of patients visiting day hospitals between March–June 2020 compared to March–June 2019 [19].

Undeniably, all the changes mentioned above may have consequences on the outcome of cancer patients, and several estimates have been made in this regard. Using data from the lockdown period, it has been predicted that a 3-month delay in diagnoses will result in a 15–18% reduction in the 10-year net survival for bladder, brain, head and neck, liver, lung, esophagus, ovary, and stomach cancer [20]. Compared to pre-pandemic data, an estimated 7.9–9.6% increase in the number of deaths due to breast cancer up to year 5 after diagnosis was determined. The increase is 15.3–16.6% for colorectal cancer, 4.8–5.3% for lung cancer, and 5.8–6% for esophageal cancer [21]. Modeling the effect of COVID-19 on cancer screening and treatment for breast and colorectal cancer in a US scenario, almost 10,000 excess deaths are expected over the next decade [3].

The information published to date offers estimates or partial data regarding the effect of the pandemic on the incidence rates of new cases of cancer, providing results only for the first months of the pandemic. The purpose of this study is to evaluate the real changes, without estimates, in the rate of cancer diagnosis during the first full year since the declaration of the lockdown in a population area of approximately one and a half million inhabitants. This study also includes all types of neoplasms.

## 2. Materials and Methods

This cross-sectional study involved all patients with a pathological diagnosis of cancer who had at least one encounter in two university-affiliated hospitals in Málaga (Spain, Hospital Regional and Hospital Virgen de la Victoria) during the first year of the pandemic. Between the two hospitals, an area of 1,500,000 people is attended, representing 80% of the total province’s population. All patients with at least one encounter in either of the two hospitals are always registered in our own information system. This system is called Galen and is comprised of a database of patients and their electronic health records, among other utilities, and includes data on more than 49,000 patients and nearly 54,000 neoplasms [22].

In Spain, the state of national emergency was declared as a result of COVID-19 on 13 March 2020. Thus, the study population considered all patients diagnosed between 13 March 2020 and 13 March 2021, and the reference population considered those cases diagnosed between 13 March 2019 and 13 March 2020, including all tumor sites. The date of the pathological report was taken as the diagnosis date. In order to clarify whether the differences found in the incidence rates between the study population and the reference group were due to the pandemic or to unrelated variations, the patients diagnosed in the same periods in the years 2018 and 2017 were also compared with the reference population. If significant differences were identified in the total incidence rate between 2020 and 2019, but no significant differences were observed between 2018 and 2019 and between 2017 and 2019, the hypothesis that the COVID-19 pandemic has significantly impaired the process of diagnosing cancer patients would be supported. Month 1 of the analysis was considered to elapse between March 13 and April 12 of the corresponding year and so on until month 12, which was considered to elapse between February 13 and March 12 of the following year. The number of COVID-19 cases diagnosed monthly in the province of Málaga was also considered when analyzing the evolution in the incidence of new cancer cases during the first year of the pandemic [23].

In addition, this study also analyzed whether there were changes in the distribution of stages of neoplasms at diagnosis in the different populations included. For this purpose, stages I-II were grouped as early stage and III-IV as advanced stage. Patients with an unknown stage were excluded for this second analysis.

Although the analysis of this study is retrospective, all the data used have been collected prospectively. The numbers of new diagnosed cancer cases in the different time periods evaluated were compared using the Wilcoxon test for paired samples. The Fisher test was used to analyze the possible differences in the distribution of stages at diagnosis between the distinct study periods. All statistical analysis was carried out using R version 3.6.1 ( R Foundation for Statistical Computing, Vienna, Austria), assuming a *p* value of 0.05. 

## 3. Results

During 2020, there were 2340 new patients diagnosed with cancer compared to 2825 in 2019, with similar median age and gender distribution in both populations (Table 1). The neoplasms with the highest incidence rates were breast, lung, colorectal, hematological, head and neck, and pancreas (Table 2). In absolute terms, 485 fewer patients were diagnosed in 2020 than in 2019, which represents a statistically significant decrease of −17.2% (*p* = 0.0001). The neoplasms with the greatest relative declines, between −47.1% and −32.4%, were CNS, kidney, prostate, mesothelioma, and gallbladder and bile duct tumors (Table 2).

In addition, it is necessary to highlight that, during 2020, 195 breast cancers (−26.1%), 82 colorectal neoplasms (−16.9%), or 24 head and neck tumors (−19.8%) were not diagnosed in comparison to 2019. However, the relative change in lung or pancreatic tumors was smaller, −5.9% and −1.1%, respectively, which meant that 29 less patients were diagnosed with lung cancer and only one less patient was diagnosed with pancreatic cancer. 

All types of neoplasms experienced a decrease in their incidence in 2020 compared to 2019, except for hematological tumors and, to a lesser extent, gastrointestinal stromal tumor (GIST) and ovarian tumors.

**Figure 1:**
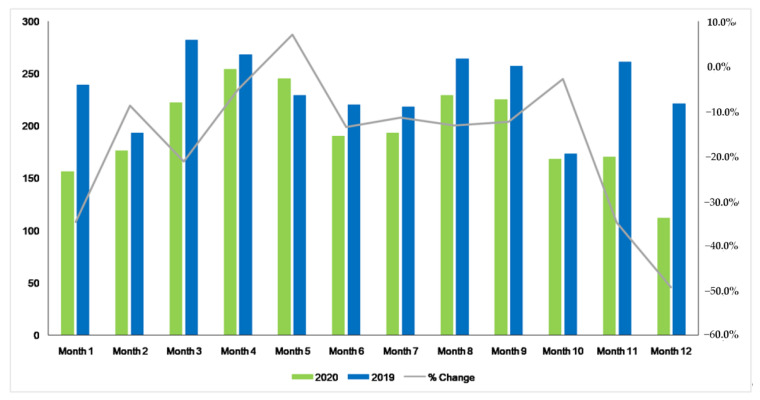
Comparative of monthly new diagnosis of all tumor sites between 2020 and 2019.

If we look at the changes observed in the monthly number of diagnoses between 2020 and 2019 (Figure 1) and we evaluate them in relation to the number of COVID-19 cases diagnosed monthly in the province of Málaga (Table S1), we can notice that, during the first month of the pandemic, there was a decrease of −34.7% in the number of diagnosed patients, which reached −34.9% and −49.3% in months 11 and 12 of the study, coinciding with the third wave after Christmas.

In 2018, 2840 new cases of cancer were diagnosed, and in 2017, there were 2909. As expected, the median age and the distribution by gender were similar for the two years and also for 2019 (Tables S2 and S3). The incident rate analysis did not show significant differences between 2018 and 2019 and between 2017 and 2019. There was an increase of 0.5% between 2018 and 2019 (P = 0.96). The increase was slightly higher between 2017 and 2019, reaching 3% (P = 0.68).

Table S4 detail the annual incidence of each type of neoplasia in 2018 and Table S5 the same data regarding 2017, as well as their relative variation with respect to 2019. In both study periods, only some of the types of neoplasms analyzed showed changes in their incidence.

The analysis of the monthly changes between 2018 and 2019 is shown in Supplementary Figure S1 and between 2017 and 2019 is presented in Supplementary Figure S2. As expected, changes are observed in the number of patients diagnosed according to the months, but the differences between the study periods never reach the magnitude of the greatest changes observed during the first year of the COVID-19 pandemic.

**Figure 2:**
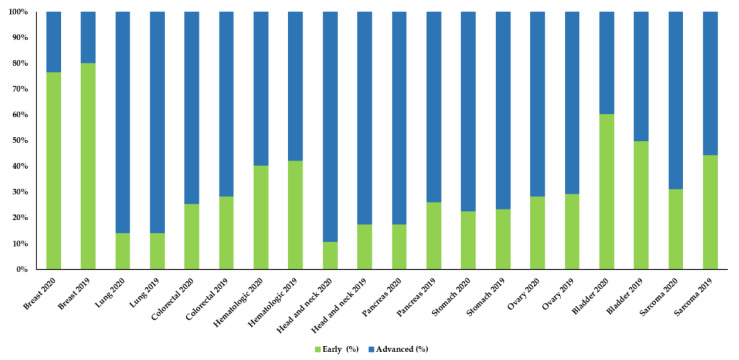
Comparison of early and advanced stages in the ten most frequent neoplasms between 2020 and 2019.

The analysis of the distribution by stages at diagnosis did not show significant differences between the number of early and advanced cases diagnosed in 2020 and 2019, nor in the other two study periods analyzed (Figure 2, Figures S3 and S4).

However, if we examine the data in detail, we can observe that there are some neoplasms, in which the percentage of advanced stages at diagnosis had remained relatively constant during the previous years (2019, 2018, and 2017), that, in 2020showed an increase in the number advanced cases. Thus, pancreatic cancer showed an increase of 8.7%, germ cells and head and neck tumors increased around 6%, and breast and colon neoplasms increased over 3% (Table 3, Tables S6 and S7).

## 4. Discussion

The appearance of the COVID-19 pandemic has resulted in serious consequences for oncology, starting with the delay in diagnostic procedures. In the UK, 35% fewer diagnostic tests were performed between March and August 2020 compared to the same period in 2019 [24]. In North Carolina (USA), the monthly number of diagnostic mammography and breast biopsies was reduced to a maximum of −48.9% and −40.9%, respectively, after March 2020 [25]. The number of colonoscopies performed in England during April 2020 was 92% lower than for the same month in 2019 [17]. These changes in the number of diagnostic procedures routinely performed have necessarily led to a general decline in the number of patients diagnosed with cancer since the beginning of the pandemic. During March and April 2020, the mean number of weekly patients diagnosed with breast, colorectal, lung, pancreatic, or gastric cancer in the US decreased by 46.6% for these 6 types of cancer combined, with the greatest reduction for breast cancer (−51.8%) and the lowest for pancreatic cancer (−24.7%) [10]. Similar findings have been reported in the number of weekly patients diagnosed during the first four months of the pandemic in the Netherlands [6], Italy [11], Germany [12], Spain [19] or Australia [26].

Our results show a significant decrease in the total number of patients diagnosed during the entire first year after the pandemic compared to the previous year. Thus, in 2020, 2340 new cancer patients were diagnosed compared to 2825 in 2019; that is, 485 (−17.2%) potential cancer patients were still undiagnosed. Although the relative decrease in the number of cases was greater in some types of neoplasms, such as CNS, kidney, prostate, or mesothelioma, the findings observed for breast and colorectal cancer are more impressive. Certainly, although the percentage of relative change in 2020 compared to 2019 has been lower in breast and colorectal cancer, their total incidence in the population determines that the situation caused by the COVID-19 pandemic negatively affects a greater number of patients. In fact, both types of neoplasms are categories in which we have observed a greater number of patients that have not been diagnosed in 2020 compared to 2019: 195 patients for breast cancer and 82 for colon cancer. It should be noted that, in these two types of neoplasms, screening programs constitute a relevant procedure in their management, and perhaps this issue may have influenced the decline in their diagnoses. Although there is no data in this regard from Spain, in other countries such as UK [7,24], the Netherlands [6], Australia [26], US [9,25], or Italy [14], screening programs have been suspended during certain periods of the pandemic.

An obvious consequence of the delay in diagnosis is that patients will present a more advanced stage of the disease when they are finally diagnosed over the next few months, which will undoubtedly worsen their prognosis, but health systems will also be greatly affected since the treatment and management of advanced cancer is much more expensive than during the early stages. There are data that indicate that the pandemic has significantly influenced the increase in advanced stages at diagnosis in melanoma [13], breast [14,15], and lung cancer [16]. In our population, we have observed an increase in advanced cases of pancreatic, germ cell, head and neck cancer, and to a lesser extent, breast and colorectal cancer. This difference has not been statistically significant, which could be due to the sample size. This could also be because not enough time has elapsed for this difference to be evident. We believe that the distribution of disease stages will change in the coming months.

The main strength of our work is that, to our knowledge, this is the first published data on the effect of the COVID-19 pandemic on cancer diagnosis for a full year that also includes all tumor types [6,10,11,12]. It should be noted that our data and comparisons are real and not estimates, and that the monthly number of diagnosed COIVD-19 cases have also been included in the analysis. However, a limitation of our study is that it does not include the entire population of the province of Málaga, as data from the other two small hospitals are not available. In addition, there are a number of private providers that deliver medical oncology whose data are not available. On the other hand, the data referring to a tumor type as relevant, such as prostate neoplasms, are not assessable since the healthcare structure of its management has undergone changes in the last 3 years.

Our study shows that a significant number of cancer patients have not been diagnosed throughout 2020, and that they would have been diagnosed if the COVID-19 pandemic had not occurred. It is true that health systems have done what needed to be done to control the COVID-19 pandemic. But it is no less true that health systems must enable all the necessary resources to improve procedures for cancer patients in order to recover all lost patients who have remained undiagnosed, as well as to prevent new patients from encountering a similar situation of diagnostic delay.

## 5. Conclusions

During the first year of the COVID-19 pandemic there has been a significant decrease in the number of cancer patients diagnosed in our area, due not only to changes in primary and hospital care, but probably also due to the fear of the patients themselves to get COVID-19 infection. It will take a great effort from the health authorities to care for these lost patients while continuing with the usual workload from 2021 onwards.

## Figures and Tables

**Table 1:** Patients’ characteristics 2020 and 2019.

Characteristic	2020	2019
N	2340	2825
Age at diagnoses		
Median (range)	63 (14–96)	64 (17–100)
Gender		
Male	1089	1324
Female	1251	1501
Early Stage		
0–0_is_	5	12
I	326	434
II	477	603
TOTAL	808	1049
Advanced Stage		
III	556	623
IV	855	939
Total	1378	1562
Unknown Stage	154	214

**Table 2:** Neoplasms annual incidence and relative changes.

Neoplasm	2020		2019		% Change
Breast	551	(23.5%)	746	(26.4%)	−26.1%
Lung	463	(19.8%)	492	(17.4%)	−5.9%
Colorectal	402	(17.2%)	484	(17.1%)	−16.9%
Hematologic	108	(4.6%)	86	(3.0%)	25.6%
Head and Neck	97	(4.1%)	121	(4.3%)	−19.8%
Pancreas	90	(3.8%)	91	(3.2%)	−1.1%
Stomach	71	(3.0%)	77	(2.4%)	−7.8%
Ovary	71	(3.0%)	69	(2.7%)	2.9%
Bladder	58	(2.5%)	70	(2.5%)	−17.1%
Sarcoma	54	(2.3%)	73	(2.6%)	−26.0%
Esophagus	48	(2.1%)	52	(1.8%)	−7.7%
Gallbladder and bile duct	41	(1.8%)	61	(2.2%)	−32.8%
Prostate	39	(1.7%)	61	(2.2%)	−36.1%
Cervix	38	(1.6%)	46	(1.6%)	−17.4%
Others	35	(1.5%)	64	(2.3%)	−45.3%
Melanoma	34	(1.5%)	41	(1.5%)	−17.1%
Germ cells	31	(1.3%)	33	(1.2%)	−6.1%
Endometrium	25	(1.1%)	37	(1.3%)	−32.4%
Kidney	22	(0.9%)	37	(1.3%)	−40.5%
GIST	19	(0.8%)	15	(0.5%)	26.7%
CNS	18	(0.8%)	34	(1.2%)	−47.1%
CUP	17	(0.7%)	23	(0.8%)	−26.1%
Mesothelioma	8	(0.3%)	12	(0.4%)	−33.3%
TOTAL ^1^	2340	(100%)	2825	(100%)	−17.2%

^1^*p* = 0.0001. CUP: Cancer of unknown primary site; GIST: Gastrointestinal stromal tumor; CNS: central nervous system.

**Table 3:** Annual distribution of tumor stages 2020–2019.

NEOPLASIAS	Early 2020	Early 2020 (%)	Early 2019	Early 2019 (%)	Advanced 2020	Advanced 2020 (%)	Advanced 2019	Advanced 2019 (%)
Breast	410	76.6%	587	80.3%	125	23.4%	144	19.7%
Lung	65	14.2%	69	14.2%	392	85.8%	416	85.8%
Colorectal	100	25.5%	135	28.5%	292	74.5%	339	71.5%
Hematologic	38	40.4%	33	42.3%	56	59.6%	45	57.7%
Head and neck	10	10.9%	19	17.6%	82	89.1%	89	82.4%
Pancreas	14	17.5%	22	26.2%	66	82.5%	62	73.8%
Ovary	19	28.4%	18	29.5%	48	71.6%	43	70.5%
Stomach	15	22.7%	16	23.5%	51	77.3%	52	76.5%
Bladder	29	60.4%	31	50.0%	19	39.6%	31	50.0%
Esophagus	5	11.6%	9	20.0%	38	88.4%	36	80.0%
Gallbladder and bile duct	7	17.9%	7	12.7%	32	82.1%	48	87.3%
Cervix	20	52.6%	22	48.9%	18	47.4%	23	51.1%
Prostate	5	14.3%	4	7.3%	30	85.7%	51	92.7%
Melanoma	15	46.9%	4	10.5%	17	53.1%	34	89.5%
Sarcoma	10	31.3%	16	44.4%	22	68.8%	20	55.6%
Germ cells	25	86.2%	25	92.6%	4	13.8%	2	7.4%
Endometrium	8	32.0%	11	30.6%	17	68.0%	25	69.4%
Others	6	24.0%	10	20.0%	19	76.0%	40	80.0%
Kidney	0	0.0%	5	14.3%	20	100.0%	30	85.7%
CUP	0	0.0%	0	0.0%	17	100.0%	23	100.0%
GIST	7	43.8%	6	60.0%	9	56.3%	4	40.0%
Mesothelioma	0	0.0%	0	0.0%	4	100.0%	7	100.0%

P = non-significant between stages at diagnosis 2020 vs 2019.

## Data Availability

The data that support the findings of this study are available on request from the corresponding author.

## Supplementary Materials

The following are available online at https://www.mdpi.com/article/10.3390/cancers13133215/s1, Figure S1: Comparative of monthly new diagnosis of all tumor sites between 2018 and 2019, Figure S2. Comparative of monthly new diagnosis of all tumor sites between 2017 and 2019, Figure S3. Comparison of early and advanced stages in the ten most frequent neoplasms between 2018 and 2019, Figure S4. Comparison of early and advanced stages in the ten most frequent neoplasms between 2017 and 2019, Table S1 Monthly number of COVID-19 cases in Málaga, Table S2 Patients’ characteristics 2018 and 2019, Table S3 Patients’ characteristics 2017 and 2019, Table S4 Neoplasms annual incidence and relative changes 2018 and 2019, Table S5 Neoplasms annual incidence and relative changes 2017 and 2019, Table S6 Annual distribution of tumor stages 2018 and 2019, Table S7 Annual distribution of tumor stages 2017 and 2019.
