# Supplementary material for: Significant Decrease in Annual Cancer Diagnoses in Spain during the COVID-19 Pandemic: A Real-Data Study
Source: Cancers (Basel). 2021 Jun 28;13(13):3215. doi: 10.3390/cancers13133215 (PMC8267675; doi:10.3390/cancers13133215)
Supplement: Supplementary file 1 [file cancers-13-03215-s001.zip › cancers-1266449-supplementary.pdf]

Table S1. Monthly number of COVID-19 cases in Málaga

MONTH	No. COVID-19 cases	% change in cancer diagnoses 2019-2020
MONTH 1	2,578	-30,4%
MONTH 2	1,528	-15,2%
MONTH 3	442	-20,7%
MONTH 4	184	-4,7%
MONTH 5	1,518	7,1%
MONTH 6	7,967	-19,0%
MONTH 7	5,918	-4,8%
MONTH 8	11,315	-14,5%
MONTH 9	6,462	-17,1%
MONTH 10	12,047	4,6%
MONTH 11	34,06	-36,7%
MONTH 12	5,178	-49,3%

Table S2. Patients' characteristics 2018 and 2019

Characteristic	2018	2019
N	2,840	2,825
Age at diagnoses		
Median (range)	64 (14-97)	64 (17-100)
Gender		
Male	1,334	1,324
Female	1,506	1,501
Early Stage		
0-0_{is}	17	12
I	414	434
II	641	603
TOTAL	1,071	1,049
Advanced Stage		
III	617	623
IV	891	939
Total	1,508	1,562
Unknown Stage	261	214

Table S3. Patients' characteristics 2017and 2019

Characteristic	2017	2019
N	2,909	2,825
Age at diagnoses		
Median (range)	63 (15-94)	64 (17-100)
Gender		
Male	1,398	1,324
Female	1,511	1,501
Early Stage		
0-0_{is}	17	12
I	414	434
II	641	603
TOTAL	1,071	1,049
Advanced Stage		
III	617	623
IV	891	939
Total	1,508	1,562
Unknown Stage	261	214

Table S 4. Neoplasms annual incidence and relative changes.

Neoplasm	2018		2019		% Change
Breast	758	(26,7%)	746	(26,4%)	1,6%
Lung	498	(17,5%)	492	(17,4%)	1,2%
Colorectal	493	(17,4%)	484	(17,1%)	1,9%
Head and Neck	115	(4,0%)	121	(4,3%)	-5,0%
Hematologic	114	(4,0%)	86	(3,0%)	32,6%
Pancreas	101	(3,6%)	91	(3,2%)	11,0%
Stomach	82	(2,9%)	77	(2,7%)	6,5%
Bladder	76	(2,7%)	70	(2,5%)	8,6%
Others	71	(2,5%)	64	(2,3%)	10,9%
Prostate	66	(2,3%)	61	(2,2%)	8,2%
Ovary	61	(2,1%)	69	(2,4%)	-11,6%
Melanoma	59	(2,1%)	41	(1,5%)	43,9%
Sarcoma	56	(2,0%)	73	(2,6%)	-23,3%
Esophagus	38	(1,3%)	52	(1,8%)	-26,9%
Endometrium	37	(1,3%)	37	(1,3%)	0,0%
Germ cells	36	(1,3%)	33	(1,2%)	9,1%
CUP	36	(1,3%)	23	(0,8%)	56,5%
Cervix	35	(1,2%)	46	(1,6%)	-23,9%
Gallbladder and bile duct	33	(1,2%)	61	(2,2%)	-45,9%
Kidney	29	(1,0%)	37	(1,3%)	-21,6%
CNS	27	(1,0%)	34	(1,2%)	-20,6%
GIST	13	(0,5%)	15	(0,5%)	-13,3%
Mesothelioma	6	(0,2%)	12	(0,4%)	-50,0%
TOTAL[1]	2,840	(100%)	2,825	(100%)	0,5%

[1]*P*=0,96. CUP: Cancer of unknown primary site GIST: Gastrointestinal stromal tumor CNS: central nervous system

Table S5. Neoplasms annual incidence and relative changes.

Neoplasm	2018		2019		% Change
Breast	786	(27,0%)	746	(26,4%)	5,4%
Lung	505	(17,4%)	492	(17,4%)	2,6%
Colorectal	480	(16,5%)	484	(17,1%)	-0,8%
Head and Neck	130	(4,5%)	121	(4,3%)	7,4%
Prostate	108	(3,7%)	61	(2,2%)	77,0%
Pancreas	92	(3,2%)	91	(3,2%)	1,1%
Hematologic	85	(2,9%)	86	(3,0%)	-1,2%
Bladder	79	(2,7%)	70	(2,5%)	12,9%
Ovary	68	(2,3%)	69	(2,4%)	-1,4%
Others	67	(2,3%)	64	(2,3%)	4,7%
Stomach	65	(2,2%)	77	(2,7%)	-15,6%
Melanoma	65	(2,2%)	41	(1,5%)	58,5%
Sarcoma	63	(2,2%)	73	(2,6%)	-13,7%
Esophagus	49	(1,7%)	52	(1,8%)	-5,8%
Gallbladder and bile duct	46	(1,6%)	61	(2,2%)	-24,6%
Cervix	43	(1,5%)	46	(1,6%)	-6,5%
CUP	37	(1,3%)	23	(0,8%)	60,9%
Germ cells	36	(1,2%)	33	(1,2%)	9,1%
Kidney	35	(1,2%)	37	(1,3%)	-5,4%
Endometrium	30	(1,0%)	37	(1,3%)	-18,9%
CNS	28	(1,0%)	34	(1,2%)	-17,6%
Mesothelioma	10	(0,3%)	12	(0,4%)	-16,7%
GIST	2	(0,1%)	15	(0,5%)	-86,7%
TOTAL[1]	2,909	(100%)	2,825	(100%)	3,0%

[1]*P*=0,68. CUP: Cancer of unknown primary site GIST: Gastrointestinal stromal tumor CNS: central nervous system

Figure- S1: Comparative of monthly new diagnosis of all tumor sites between 2018 and 2019

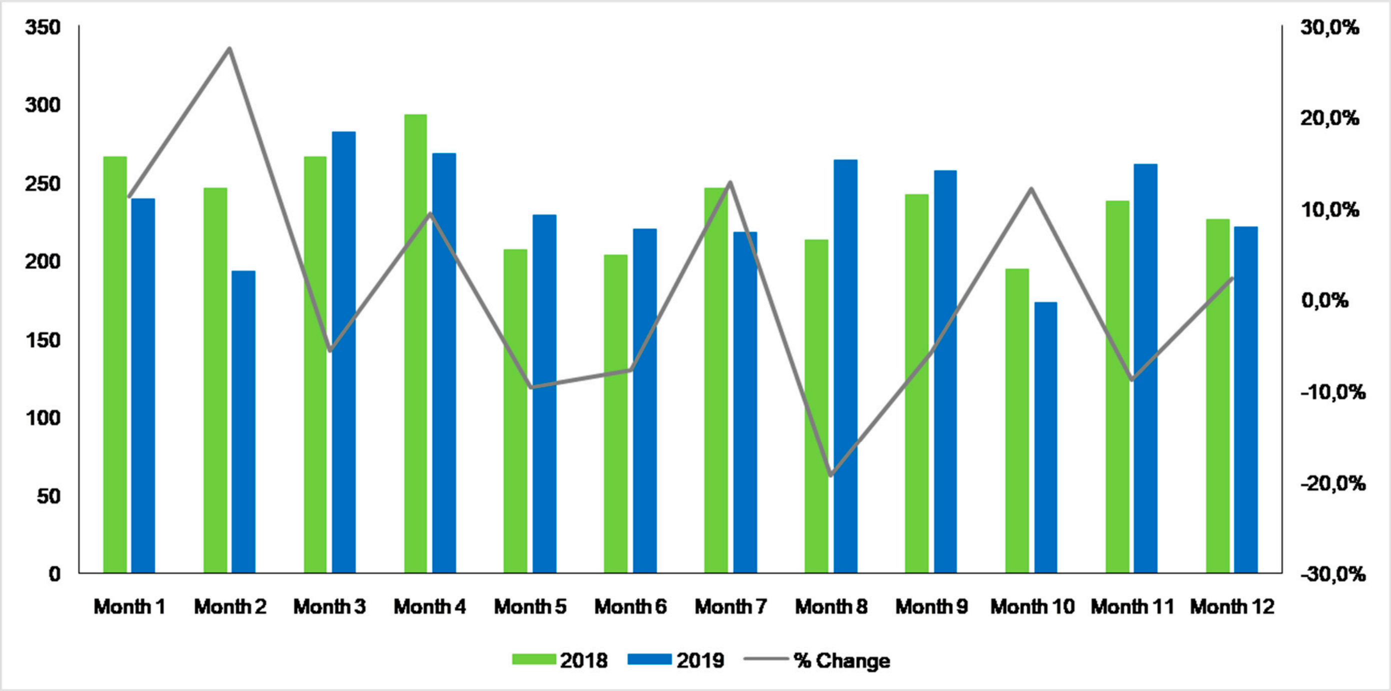

Figure- S2: Comparative of monthly new diagnosis of all tumor sites between 2017 and 2019

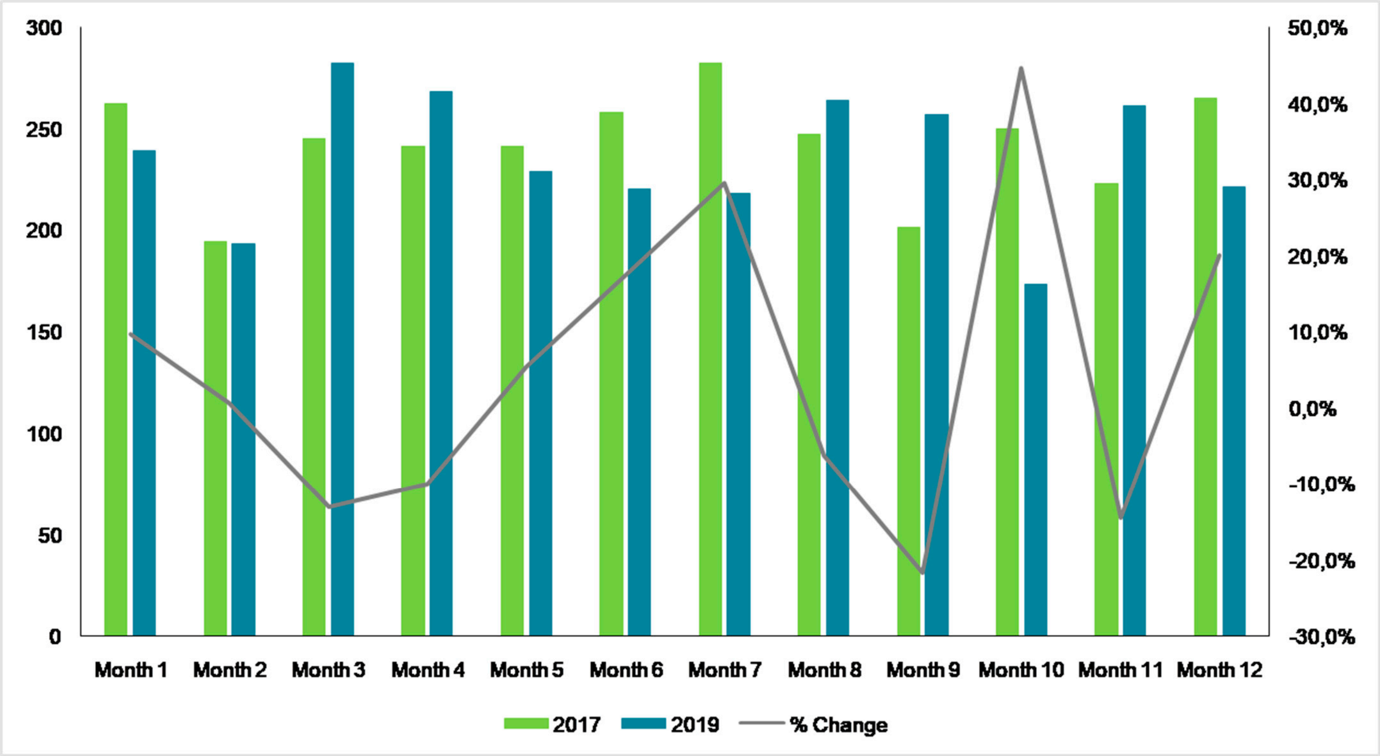

Figure S3. Comparison of early and advanced stages in the ten most frequent neoplasms between 2018 and 2019

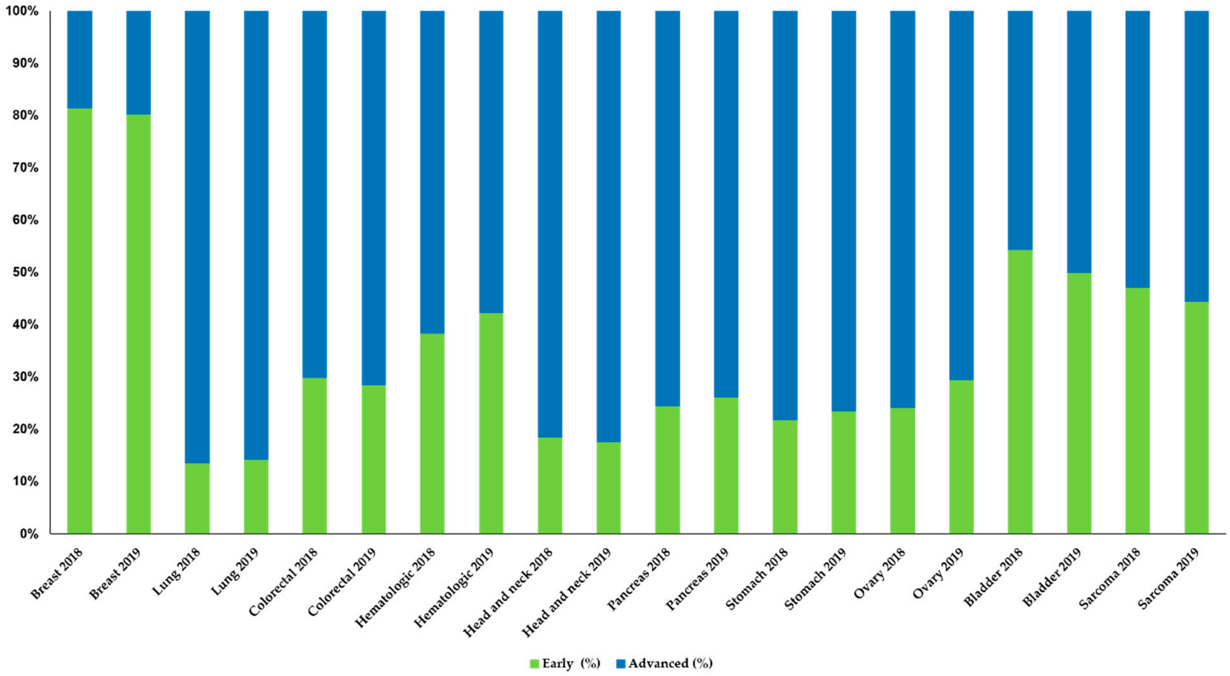

Figure S4. Comparison of early and advanced stages in the ten most frequent neoplasms between 2017 and 2019

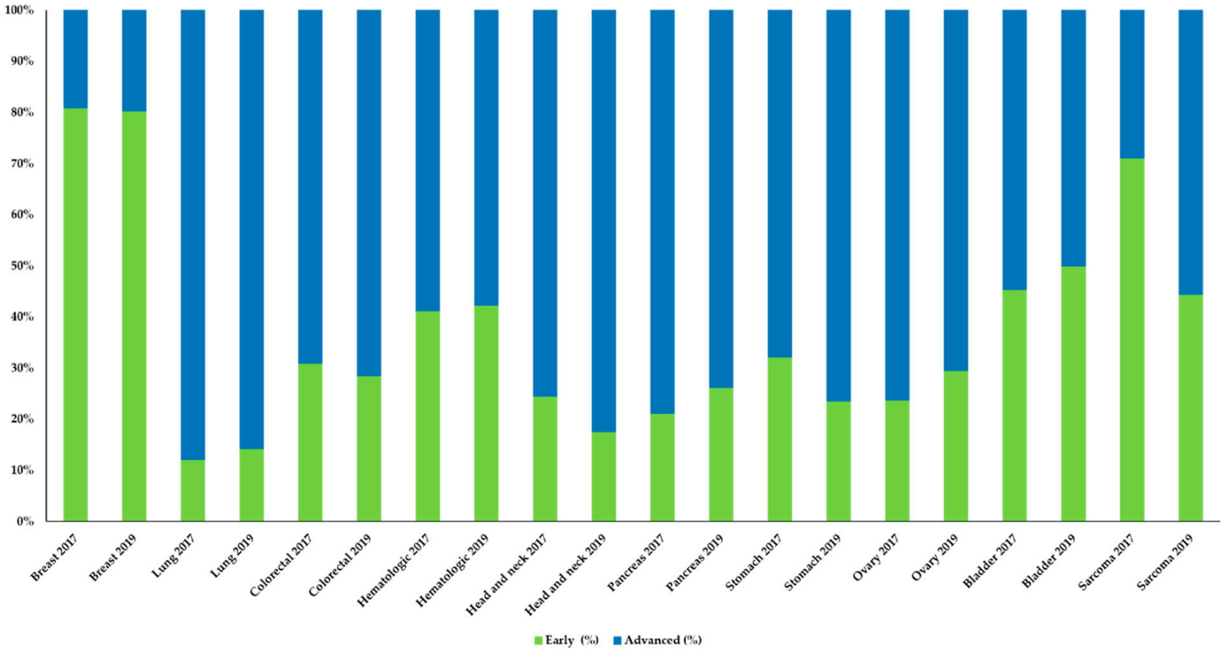

Table S6. Annual distribution of tumor stages 2018 and 2019

NEOPLASIAS	Early 2018	Early 2018 (%)	Early 2019	Early 2019 (%)	Advanced 2018	Advanced 2018 (%)	Advanced 2019	Advanced 2019 (%)
Breast	586	81,4%	587	80,3%	134	18,6%	144	19,7%
Lung	65	13,5%	69	14,2%	415	86,5%	416	85,8%
Colorectal	143	30,0%	135	28,5%	334	70,0%	339	71,5%
Hematologic	38	38,4%	33	42,3%	61	61,6%	45	57,7%
Head and neck	18	18,6%	19	17,6%	79	81,4%	89	82,4%
Pancreas	23	24,5%	22	26,2%	71	75,5%	62	73,8%
Ovary	13	24,1%	18	29,5%	41	75,9%	43	70,5%
Stomach	17	21,8%	16	23,5%	61	78,2%	52	76,5%
Bladder	31	54,4%	31	50,0%	26	45,6%	31	50,0%
Esophagus	3	11,1%	9	20,0%	24	88,9%	36	80,0%
Gallbladder and bile duct	8	27,6%	7	12,7%	21	72,4%	48	87,3%
Cervix	20	57,1%	22	48,9%	15	42,9%	23	51,1%
Prostate	8	13,3%	4	7,3%	52	86,7%	51	92,7%
Melanoma	12	27,9%	4	10,5%	31	72,1%	34	89,5%
Sarcoma	17	47,2%	16	44,4%	19	52,8%	20	55,6%
Germ cells	31	93,9%	25	92,6%	2	6,1%	2	7,4%
Endometrium	11	32,4%	11	30,6%	23	67,6%	25	69,4%
Others	13	27,1%	10	20,0%	35	72,9%	40	80,0%
Kidney	8	28,6%	5	14,3%	20	71,4%	30	85,7%
CUP	0	0,0%	0	0,0%	36	100,0%	23	100,0%
GIST	5	50,0%	6	60,0%	5	50,0%	4	40,0%
Mesothelioma	1	25,0%	0	0,0%	3	75,0%	7	100,0%

P=non-significant

Table S7. Annual distribution of tumor stages 2017 and 2019

NEOPLASIAS	Early 2017	Early 2017 (%)	Early 2019	Early 2019 (%)	Advanced 2017	Advanced 2017 (%)	Advanced 2019	Advanced 2019 (%)
Breast	601	80,9%	587	80,3%	142	19,1%	144	19,7%
Lung	58	12,2%	69	14,2%	418	87,8%	416	85,8%
Colorectal	142	30,9%	135	28,5%	317	69,1%	339	71,5%
Hematologic	28	41,2%	33	42,3%	40	58,8%	45	57,7%
Head and neck	29	24,6%	19	17,6%	89	75,4%	89	82,4%
Pancreas	18	21,2%	22	26,2%	67	78,8%	62	73,8%
Ovary	15	23,8%	18	29,5%	48	76,2%	43	70,5%
Stomach	19	32,2%	16	23,5%	40	67,8%	52	76,5%
Bladder	30	45,5%	31	50,0%	36	54,5%	31	50,0%
Esophagus	13	28,9%	9	20,0%	32	71,1%	36	80,0%
Gallbladder and bile duct	4	12,9%	7	12,7%	27	87,1%	48	87,3%
Cervix	26	61,9%	22	48,9%	16	38,1%	23	51,1%
Prostate	13	13,7%	4	7,3%	82	86,3%	51	92,7%
Melanoma	20	35,7%	4	10,5%	36	64,3%	34	89,5%
Sarcoma	32	71,1%	16	44,4%	13	28,9%	20	55,6%
Germ cells	33	91,7%	25	92,6%	3	8,3%	2	7,4%
Endometrium	12	42,9%	11	30,6%	16	57,1%	25	69,4%
Others	9	19,1%	10	20,0%	38	80,9%	40	80,0%
Kidney	6	20,0%	5	14,3%	24	80,0%	30	85,7%
CUP	1	2,8%	0	0,0%	35	97,2%	23	100,0%
GIST	5	83,3%	6	60,0%	1	16,7%	4	40,0%
Mesothelioma	2	22,2%	0	0,0%	7	77,8%	7	100,0%

P=non-significant
